# The Role of S100b Protein Biomarker in Brain Death: A Literature Review

**DOI:** 10.7759/cureus.62707

**Published:** 2024-06-19

**Authors:** Anderson N Lopes, Andrea Regner, Daniel Simon

**Affiliations:** 1 Critical Care, Universidade Luterana do Brasil, Canoas, BRA; 2 Critical Care, Hospital Materno Infantil Presidente Vargas, Porto Alegre, BRA; 3 Genetics, Universidade Luterana do Brasil, Canoas, BRA

**Keywords:** brain injuries, stroke, traumatic brain injuries, biomarker, s100 beta, brain death

## Abstract

Brain death (BD) represents the irreversible loss of all brain functions, including the brainstem, and is equivalent to clinical death established by neurological criteria. However, clinical diagnosis, mainly based on the absence of primary reflexes post-acute brain injury, remains a challenge in hospital settings. The S100 calcium-binding protein beta (S100b) is used to monitor brain injuries, as recommended by neurotrauma care guidelines in some countries. Its levels are associated with severity and mortality, particularly after traumatic brain injury (TBI) and cerebral hemorrhage. The evaluation of S100b levels in investigating brain death is promising; however, aspects such as cutoff values remain to be elucidated. This paper reviews the literature on the use of S100b as a biomarker in diagnosing brain death. It is noteworthy that there is still no defined cutoff for S100b levels in confirming brain death. Additionally, when considering the use of S100b in emergency situations, a point-of-care methodology should be established to support clinical decision-making quickly and easily in the early identification of patients who are more likely to progress to brain death. In this context, S100b levels may assist in establishing the diagnosis of brain death, complementing existing clinical evidence. This, in turn, can optimize and qualify the organ donation process, reducing costs with ineffective therapies and minimizing the suffering of the families involved.

## Introduction and background

Brain death (BD) represents cerebral and brainstem death, equivalent to clinical death established by neurological criteria [1]. Irreversible brainstem injury is compatible with death in modern intensive care units (ICUs); however, challenges still exist in its diagnosis [2]. BD typically results from acute brain injury, especially due to traumatic brain injury (TBI) and hemorrhagic stroke, but can also result from extracranial causes such as cardiopulmonary arrest without appropriate resuscitation [2].

Despite well-established criteria for confirming BD, the understanding of organic changes and their course over time are not fully elucidated [1-3]. After the occurrence of acute brain damage, the progression of primary neural injury forms areas of penumbra that may evolve with cellular distress culminating in death or, alternatively, may trigger adaptive reactions in an effort toward neurorestoration [4]. The final progression of cellular injury evolves with the rostrocaudal progression of ischemia, resulting in uncontrollable intracranial pressure (ICP) and BD. This evolution involves structures of the skull base such as the midbrain, pons, and medulla, resulting in a process of brain herniation through the foramen magnum [1]. In this catastrophic scenario, the blood-brain barrier becomes vulnerable and permissive to the passage of molecules released by cell death, which are thus released into systemic circulation. The investigation of potential plasma biomarkers for the diagnosis of BD, in conjunction with the clinical criteria established by BD legislation, may provide the healthcare team with more elements to expedite and qualify the process of clinical decision-making, thereby shortening the time to BD diagnosis and reducing the use of ineffective therapies, allowing better viability of future organs and tissues available for transplantation, with family consent. BD is also related to organ and tissue donation, a procedure that is a significant advancement in the treatment of diseases of renal, hepatic, cardiac, and pulmonary origin. However, globally, a difficulty faced with regard to transplants is the much greater demand than the supply of organs [5] and the underreporting of BD cases, from which most solid organs for transplantation originate [6].

Although the predictive role of plasma biomarkers in acute neural injury is well established [3,7-10], the BD diagnostic scenario leads us to another reality: scarcity of evidence of the role of circulating biomarkers as diagnostic tools to aid in clinical decision-making, indication of patient inclusion in the BD protocol, and for confirmation of BD diagnosis [11]. In this context, the S100 calcium-binding protein beta (S100b) has shown consistency as a predictive molecule in various acute neural injury scenarios. This review focuses on analyzing the prognostic utility of S100b protein after acute brain injuries (particularly TBI and stroke) that progress to BD.

## Review

Brain death

Currently, BD accounts for 2% of deaths in the United States, predominantly in traumatic brain injury situations [12]. In deaths resulting from out-of-hospital cardiac arrest, one in six occurs due to irreversible brain injury [6]. The first definition of irreversible coma was established in 1968 by the Committee of the Harvard Medical School to Examine the Definition of Brain Death, which proposed this condition as death [13]. Since then, different countries around the world have adopted specific criteria for defining the diagnosis of BD [1,14-16]. The examinations for confirming BD are clinical and complementary, and directed and objective to assist medical professionals in neurological diagnosis and ensure that no confounding factors are present [1]. Although variations in the criteria adopted by protocols occur regionally, patients with clinical evidence of absence of brainstem function should be carefully evaluated for the causal event, and with confounding factors ruled out, they should be included in a protocol for confirming BD [17].

Among the main causes of irreversible brain injury are TBI and stroke, both recognized for their high rates of morbidity and mortality, resulting in high economic, social, and human costs [18-20]. TBI can be caused by various mechanisms of injury, including traffic accidents, violence, and falls from height. Mortality from traumatic brain injury is high, resulting in many years of lost life and distributed across all age groups but peaking in the first decades of life, in mostly previously healthy individuals [21]. Severity stratification in TBI is performed by applying the Glasgow Coma Scale (GCS), which quantifies the patient's level of consciousness. Based on the GCS, TBI is stratified into mild (score 14-15), moderate (9-13), and severe (3-8) [22]. However, the GCS has limitations [23]. Authors point to the difficulties of precise evaluation of the GCS in emergency scenarios, which can sometimes be chaotic [24]. It is important to note that age, GCS score, and pupillary assessment are the main predictors of outcome in traumatic brain injury [25].

Stroke is the second leading cause of all deaths and the third leading cause of disability worldwide [26]. Deaths from stroke, stratified by age, have decreased in recent decades. However, stroke remains on the priority list of the World Health Organization (WHO) for reducing the burden of non-communicable diseases [27]. Stroke is divided into two main groups: ischemic stroke (representing more than 80% of cases), defined as all atherosclerotic and thromboembolic events resulting in compromised blood flow to brain tissue and subsequent tissue infarction, and hemorrhagic stroke, defined as all non-traumatic events due to hemorrhage identified by neuroimaging and resulting in altered neural perfusion [26,27]. The mortality of hemorrhagic stroke can be higher than 30% [28].

S100B: Biomarker of central nervous system injury

The protein S100b, first described in 1965 [29], belongs to the S100 protein family, consisting of 21 proteins that exhibit different expressions in various body tissues. S100b is an abundant soluble protein in glial cells, melanocytes, adipocytes, and chondrocytes, with biological functions related to calcium regulation. Other functions of S100b were discovered after its description, such as binding to other ions (such as zinc), learning, memory, and neuroplasticity [30]. Additionally, this protein can stimulate cell proliferation, inhibit apoptosis, and promote tissue differentiation where it is present. Thus, its role may have physiological importance in processes such as development, repair, regeneration, and activation of central nervous system (CNS) cells, both in neurodegenerative conditions and acute brain injuries [31]. In TBI, with the rupture of the blood-brain barrier, alterations in cerebral blood flow in injured areas, and extravasation of proteins into the cerebrospinal fluid and through the glymphatic system, S100b reaches circulating blood in proportions 10-100 times above normal values [32]. The half-life of S100b in plasma is two hours, undergoing renal metabolism and urinary excretion [33]. In 1995, S100 was described as a serum biomarker with prognostic value in mild TBI [34]. Values between 2.0 and 2.5 µg/L were described as capable of predicting an unfavorable outcome [35]. Subsequently, S100b has been shown to be a promising biomarker for severity stratification and prediction of clinical outcomes in TBI, stroke (ischemic and hemorrhagic), and cardiac arrest [35-38]. Through automated assays (Liaison XL®, DiaSorin, and Cobas®, Roche Diagnostics) showing good reliability and reproducibility [39], S100b is already used in clinical practice [40]. Scandinavian countries were the first to include the determination of S100b levels in TBI care guidelines [41,42], and due to its excellent negative predictive value, S100b has been an alternative choice to computed tomography (CT) for monitoring therapeutic responses in managing patients with mild TBI [43].

The hypothesis that increased S100b levels predict early diagnosis of BD was described in a 2001 study by our research group when higher levels of the protein were found in severe TBI patients who progressed to BD compared to those who survived [37]. A previous study had described three patients in whom an excessive increase in S100b followed BD within 72 hours, even with controlled intracranial pressure, indicating significant secondary neural damage undetectable by available neuromonitoring technologies at the time [33].

Despite these promising results, few studies have expanded the investigation of S100b as a biomarker for predicting BD. A prospective study investigated the temporal release pattern of S100b in TBI patients admitted to the ICU [44]. In this study, it was observed that patients who progressed to BD had higher S100b concentrations than survivors. It is important to note that S100b protein does not show significant variation in relation to the age and sex of patients [45]. Furthermore, this biomarker has shown prognostic utility in pediatrics, a context in which the diagnosis of BD is more complex due to children's communication peculiarities and CNS immaturity [35].

Later, two publications from the same group of researchers in Spain investigated S100b in the BD scenario. One study proposed that S100b levels could be used as a confirmatory test for BD. It was also observed that the mean serum S100b levels in the BD group were higher than those observed in the survivor and isolated TBI groups [46]. The other study, from the same Spanish research group, included 140 patients admitted to the neurocritical care unit due to severe TBI. Clinical variables such as age, sex, GCS score, pupillary changes on admission, presence of hypotension and desaturation, CT results, isolated TBI or polytrauma, and serum S100b levels on admission, 24 hours after admission, and at the time of BD determination were evaluated. The results showed that of the 140 patients studied, 16 progressed to BD, and the absence of pupillary reaction on admission combined with elevated S100b levels in the first 24 hours after admission had a positive predictive value in diagnosing BD, suggesting that this molecule could be used in the confirmatory process of BD determination [11]. It should be noted that age was not considered a predictor for patient outcome [44]; however, there is usually a higher prevalence of males in cases of BD [11,44,46].

In a review from 2017 [47], the authors consider the applicability of S100b protein as a biomarker in different clinical scenarios, such as stratification and discharge destination in patients with mild TBI, as well as the need for CT in this patient group, prognostic prediction in patients with moderate to severe TBI, progression of secondary injuries, and monitoring of therapeutic strategies in patients with acute brain injuries. These authors suggest that the clinical use of S100b in determining BD is not recommended due to its low specificity for BD, and other tissue sources should be further explored [47]. However, one possibility would be to investigate very high cutoff points for S100b, greatly increasing the specificity of S100b for predicting BD, and since this biomarker is early, it could result in a point-of-care test in the early hours post-acute brain injury. To explore other sources, a prospective study was conducted with 83 patients with TBI or intracranial hemorrhage, comparing S100b levels in jugular venous and peripheral blood. The findings showed higher levels in the jugular vein blood sample in cases where BD occurred, reinforcing the cerebral origin of S100b [44]. These results corroborate with previous data [46], suggesting that the protein is more accurate in cases of isolated TBI. Furthermore, a study of patients with cardiogenic and septic shock undergoing extracorporeal membrane oxygenation (ECMO) treatment showed that patients with cerebral complications had increased S100b levels, regardless of whether they were receiving ECMO treatment or not [48].

The potential limitations pointed out by Thelin et al. [47] are important and should encourage further studies. In our understanding, the patient's clinical condition should be the focus of evaluation; however, early (up to 24 hours) S100b levels could complement other complementary examinations in patients with acute brain injury, supporting clinical decision-making regarding the monitoring of neurocritical patients and prompt diagnosis of BD. There is controversy in the literature regarding the S100b cutoff values that could be used for predicting BD. Bloomfield et al. [49] argue that S100b elevations above certain levels can safely predict irreversible cessation of brain and brainstem function, but there is still no consensus on the cutoff values to be adopted for the affirmative diagnosis of BD in the context of acute brain injuries.

Increased levels of S100b can be observed not only in TBI and stroke but also in various other conditions, including brain tumors, CNS infections such as encephalitis and meningitis, and neurological diseases such as multiple sclerosis and epilepsy [50]. The release of S100b from extracranial sources, such as in the case of extracranial fractures, is also a concern. Therefore, these other conditions that can elevate S100b levels may pose a challenge in interpreting S100b as a biomarker in BD. Additionally, there is variation in S100b measurement results due to different platforms used. This was evidenced by a meta-analysis that assessed S100b as a predictor of abnormalities on CT imaging following mild TBI, which reported a significant increase in sensitivity estimates when the analysis was limited to a single assay [51]. In this context, the development of point-of-care testing for S100b that is easy to use, readily available, and low-cost and provides rapid response times is needed [52,53]. Table 1 summarizes studies that have investigated circulating levels of S100b as a biomarker for predicting BD.

**Table 1:** Summary of studies analyzing circulating levels of S100b in patients with brain death outcome BD: brain death, BP: blood pressure, ECMO: extracorporeal membrane oxygenation, GCS: Glasgow Coma Scale, ICP: intracranial pressure, ICU: intensive care unit, IQR: interquartile range, OR: odds ratio, TBI: traumatic brain injury, CI: confidence interval, ELISA: enzyme-linked immunoassay

Authors (year)	Country	Number of patients	Study design/patients	Sample collected/assay employed	Results
Song et al. (2022) [6]	South Korea	253	Retrospective study including patients with out-of-hospital cardiac arrest (n=253) undergoing targeted temperature control; patients with in-hospital death (n=121) were divided into two subgroups: BD (n=19) and clinical death (n=102)	Serum obtained immediately after return of spontaneous circulation, and 24, 48, and 72 hours thereafter/electrochemiluminescence technique	S100B was higher (in the sample 72 hours after admission) in patients who progressed to BD. The median S100b levels in BD were significantly higher than those in the non-BD group (10.4 (IQR: 0.4-16.5) ng/mL versus 0.9 (IQR: 0.3-4.3) ng/mL, p=0.040).
Egea-Guerrero et al. (2013) [11]	Spain	140	Prospective cohort, patients with severe TBI who progressed (n=16) or did not progress (n=124) to brain death	Serum collected upon admission to the neuro-ICU (within 6 hours after TBI), 24 hours later, and at the time of BD determination/electrochemiluminescence technique	S100b levels were higher at admission (0.683 µg/L) and 24 hours later (0.474 µg/L) (p<0.001) in patients with BD diagnosis. For every 1 µg/L increase in S100b at admission, the OR for BD was 1.99 (95% CI: 1.21-3.32; p=0.008). In the 24-hour sample, the OR for BD was 5.37 (95% CI: 1.85-15.59; p=0.002).
Dimopoulou et al. (2003) [35]	Greece	47	Prospective cohort, severe TBI, 17 patients progressed to BD	Plasma obtained upon admission to the ICU (first sample collected 4 hours after the event) and daily until BD confirmation or the sixth day of hospitalization/immunoluminometric technique	Median levels of S100b at admission in BD and non-BD were 2.32 µg/L and 1.04 µg/L, respectively (p=0.0028). Logistic regression showed that S100b was an independent predictor of BD, with an OR of 2.09 (95% CI: 1.03-4.25) for deterioration to BD.
Regner et al. (2001) [37]	Brazil	25	Prospective cohort of severe TBI (n=15) compared with BD due to hemorrhagic stroke (n=5) and healthy controls (n=5)	Serum collected in the ICU 12-36 hours after hospital admission/immunoradiometric technique	S100b levels significantly increased in TBI patients (5.02 µg/L) and in patients with BD (5.22 µg/L). S100b was a biomarker for death, regardless of the cause (TBI or hemorrhagic stroke).
Ballesteros et al. (2018) [44]	Spain	83	Prospective study with patients suffering from TBI (n=40) and hemorrhagic stroke (n=43)	Serum collected from the jugular vein and peripheral vein upon admission to the ICU, and at 24, 48, and 72 hours thereafter/electrochemiluminescence technique	A total of 25 patients progressed to BD (14 within the first 72 hours). There was a difference in the levels of S100b collected from the jugular vein and the peripheral vein, with higher levels in the jugular vein. The transcranial gradient (the difference between the levels of S100b from the jugular vein and the peripheral vein) was significantly greater upon admission to the ICU in patients who progressed to BD.
Egea-Guerrero et al. (2013) [46]	Spain	168	Prospective cohort (n=26): 11 with severe TBI-related BD, 10 with hemorrhagic stroke, and 5 with other causes; S100b was compared to the highest value (within the first 5 days of hospitalization) from a prospective cohort (n=124) of severe TBI survivors; healthy donors (n=18) were also studied	Serum obtained immediately after confirmation of brain death/electrochemiluminescence technique	BD patients had higher S100b levels (1.44 µg/L) than the severe TBI survivor group (0.34 µg/L) and the healthy donor group (0.06 µg/L) (p<0.001). For a 1 µg/L increase in S100b value, the OR for BD diagnosis was 8.38 (95% CI: 1.16-60.45; p=0.035).
Nguyen et al. (2013) [48]	Belgium	32	Prospective cohort, patients with shock (cardiogenic and septic) treated (n=15) or not (n=17) with ECMO	Serum obtained over 5 days/immunoradiometric technique	High S100b levels were found in 75% of patients upon admission to the ICU. Patients who developed brain complications (n=5), including one with BD, had higher S100b levels on the 5th day compared to patients without brain complications (0.426 µg/L versus 0.102 µg/L; p=0.011).
Undén et al. (2004) [54]	Sweden	01	Case study of a 22-year-old patient with polytrauma and initial GCS of 3	Serum upon admission to the neuro-ICU, 24 hours later, and then every hour in response to increased ICP and BP; sample collection continued during the organ retrieval surgery for donation/chemiluminescence technique in an automated system	The S100b level upon admission was 2.4 µg/L, varying with ICP. Peak of 3.3 µg/L at 46 hours after hospital admission, when ICP reached a maximum of 120 mmHg. During the surgery for organ removal, S100b levels remained stable starting an upward curve at thoracotomy, reaching a peak of 1.9 µg/L five minutes before the heart stopped.
Shakeri et al. (2013) [55]	Iran	72	Severe isolated TBI (42 survivors and 30 non-survivors); the deceased group was divided into subgroups: clinical death (n=14) and BD (n=16)	Serum collected at hospital admission, 48 hours and 7 days later, or at the time of BD confirmation/ELISA technique	GCS at admission and the last GCS had predictive value in confirming brain death (p<0.0005). A negative correlation was observed between GCS and S100b. Patients with BD present significantly higher mean levels of S100b (2.36±0.94 µg/L) compared to patients with clinical death (1.46±0.51 µg/L) or those who survive (1.04±0.5 µg/L).

## Conclusions

The investigation of circulating biomarkers that may assist in the early and effective diagnosis of BD represents an expanding research field. In this context, the protein S100b is a promising circulating biomarker for the diagnosis of BD, as its detection in acute brain injuries, in neurocritical patients in emergency rooms and ICUs, has been employed in clinical practice for monitoring these patients in many countries. S100b is an easily obtainable biomarker, as it can be analyzed early from peripheral blood. Quantification of S100b upon hospital admission may aid in earlier diagnosis, supporting clinical decision-making by the healthcare team, avoiding ineffective therapies and family suffering, and enhancing the organ donation process. However, for such application in clinical practice, determining S100b cutoff values and establishing maximum specificity levels for BD require further studies, especially multicenter ones involving heterogeneous populations.

Maintaining a patient in irreversible coma in an ICU requires complex care and entails high costs. In this scenario, the utilization of validated, rapid, simple, and inexpensive methods to assist in the diagnosis of BD can have a significant impact. Quantification of S100b through a simple and fast method may assist medical professionals in the decision-making process to include a patient in a BD diagnosis protocol, a practice that is often delayed, resulting in reduced organ viability in potential donors and increased family suffering.
